# Imprinted Genes Impact Upon Beta Cell Function in the Current (and Potentially Next) Generation

**DOI:** 10.3389/fendo.2021.660532

**Published:** 2021-04-27

**Authors:** Chelsie Villanueva-Hayes, Steven J. Millership

**Affiliations:** Section of Cell Biology and Functional Genomics, Department of Metabolism, Digestion and Reproduction, Imperial College London, London, United Kingdom

**Keywords:** genomic imprinting, methylation, beta cell function, type 2 diabetes, diet, nutritional regulation, pancreatic islets

## Abstract

Beta cell failure lies at the centre of the aetiology and pathogenesis of type 2 diabetes and the epigenetic control of the expression of critical beta cell genes appears to play a major role in this decline. One such group of epigenetically-controlled genes, termed ‘imprinted’ genes, are characterised by transgenerational monoallelic expression due to differential allelic DNA methylation and play key functional roles within beta cells. Here, we review the evidence for this functional importance of imprinted genes in beta cells as well as their nutritional regulation by the diet and their altered methylation and/or expression in rodent models of diabetes and in type 2 diabetic islets. We also discuss imprinted genes in the context of the next generation, where dietary overnutrition in the parents can lead to their deregulation in the offspring, alongside beta cell dysfunction and defective glucose handling. Both the modulation of imprinted gene expression and the likelihood of developing type 2 diabetes in adulthood are susceptible to the impact of nutritional status in early life. Imprinted *loci*, therefore, represent an excellent opportunity with which to assess epigenomic changes in beta cells due to the diet in both the current and next generation.

## Introduction

The term “epigenetics” has been redefined frequently since the 1940s, and therefore, we will use this term to define “the study of molecules and mechanisms that can perpetuate alternative gene activity states in the context of the same DNA sequence” (1). Epigenetic mechanisms control genetic information whilst unaltering the underlying DNA sequence (2) and include DNA methylation, chromatin remodelling, histone modifications, and gene regulation by non-coding RNA. Moreover, these epigenetic pathways modulate expression of target genes, and therefore, have a significant role in the establishment, maintenance and dynamic changes in the cell (3). In mammalian genomes, DNA methylation usually refers to methylation of 5’-cytosines within CpG dinucleotides (4) and is the major pathway controlling several epigenetic phenomena, including genomic imprinting, X chromosome inactivation and repression of transposable elements (3). CpG methylation is carried out by a family of DNA methyltransferases (DNMTs) [reviewed in (5, 6)] at key regulatory genomic regions, e.g. promoters, and is associated with activation and repression of gene expression (7–13).

The epigenetic phenomenon of genomic imprinting results in monoallelic and parent-of-origin-specific gene expression in a select group of genes (14, 15). The discovery of inconsistencies in the level of DNA methylation at the same locus between paternal and maternal alleles revealed the involvement of epigenetic alterations in the regulation and conservation of monoallelic silencing in genomic imprinting (16–18). Nuclear transplantation experiments showed that embryos containing one set of parental chromosomes (uniparental disomy; UPD) did not survive beyond early gestation, demonstrating that the parental genomes were not functionally equal (19–23). Imprinting is a highly conserved process in mammals and to date, approximately 150 imprinted genes are known in mice and around 100 in humans. Imprinted genes are generally found in clusters throughout the genome and have been found to be regulated by discrete DNA elements called imprinting control regions (ICR), which are a differentially methylated region (DMR) (14, 17). Parental allele-specific imprinting marks are preserved during the lifespan (24–26), [reviewed in (27, 28)] and are reset and re-established transgenerationally (29). Secondary ‘somatic’ imprints can also be established post-fertilisation and are believed to reinforce the allele-specific gene repression at imprinted *loci* (30).

## Imprinted Genes Play Key Functional Roles in Pancreatic Beta Cells

**Figure 1:**
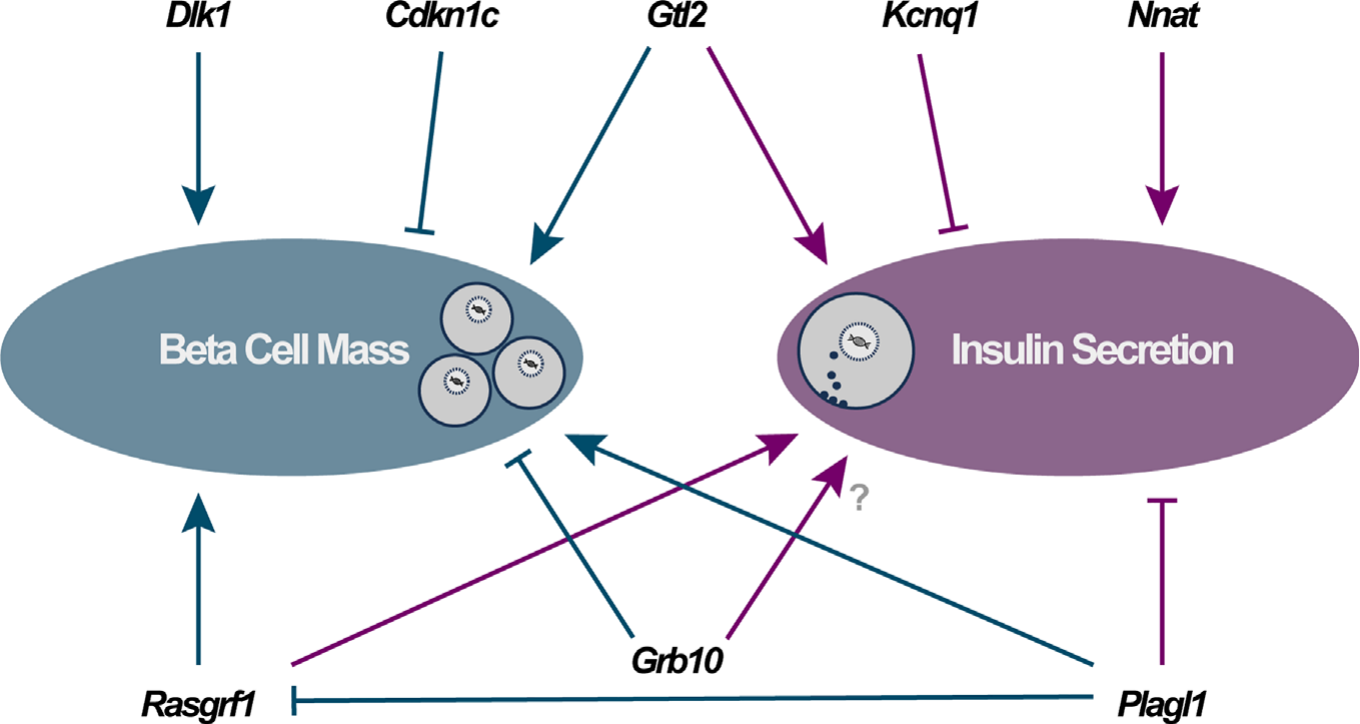
Direct functional importance of imprinted genes in pancreatic beta cells falls into two major categories: modulators of beta cell mass (via changes in cellular proliferation, apoptosis and/or differentiation) and alterations to specific components of the insulin secretory apparatus. Arrowheads and blocked lines represent stimulatory and inhibitory actions on these cellular pathways, respectively.

Imprinted genes are highly expressed in metabolic systems where they play a central role in controlling growth, development and metabolism (31). Pancreatic beta cells express a number of imprinted genes that are critical for beta cell function. Demonstrating the importance of maintaining the correct dosage of imprinted gene expression are the presence of several human imprinting syndromes (Prader-Willi, Angelman, Beckwith-Wiedemann and Silver-Russell) that result in severe developmental and metabolic abnormalities, due to altered imprinted gene expression at imprinted *loci* (15, 32, 33) [reviewed in (34)], including a transient form of neonatal diabetes caused by paternal UPD of the 6q24 region (35–37). Here, we discuss several imprinted genes with known function in beta cells (**Figure 1**), primarily through their characterisation in cellular or mutant mouse models. As a significant decline in beta cell function often coincides with a reduction in whole-body glucose homeostasis, we also discuss imprinted genes in the context of their nutritional regulation by the diet and the evidence for altered imprinted gene expression in type 2 diabetes (T2D). Finally, we explore what is known regarding susceptibility to diabetes in the next generation *via* epigenetic changes in the offspring due to parental under- or overnutrition.

### Nnat

*Nnat* is a paternally expressed imprinted gene which is highly enriched in neuroendocrine systems, including pancreatic beta cells (38–40). Early *in vitro* work reported a potential role in glucose-stimulated insulin secretion (GSIS) in two different stable mouse pancreatic beta cell lines and expression of both known isoforms of neuronatin, *Nnat-*α and *Nnat-*β, found predominantly in the endoplasmic reticulum (ER), were increased after acute stimulation with high glucose (41, 42). It has been postulated that NNAT plays a role in the regulation of the intracellular calcium dynamics in several cell types (42–44); however, primary islets from *Nnat*-null mutant mice displayed unaltered Ca^2+^ signalling (45). Both global and beta cell-specific *Nnat* deficient mice demonstrate impaired GSIS due to reduced beta cell insulin content (45). Furthermore, NNAT was shown to interact with the signal peptidase complex (SPC) and facilitates the translocation of nascent preproinsulin into the ER (45). *Nnat* expression is also regulated by nutrient status in pancreatic beta cells both *in vitro* and *in vivo* (42, 45) and in rodent models of diabetes (42, 45, 46).

### Plagl1

*Plagl1 (also known as Zac1)* is a zinc finger transcription factor that is implicated in anti-proliferative activities such as the regulation of cell cycle arrest and apoptosis (47–51). *PLAGL1* is a paternally expressed imprinted gene on chromosome 6q24, a region where paternal duplication or loss of methylation at the *PLAGL1* DMR causes transient neonatal diabetes mellitus (TNDM) locus owing to *PLAGL1* overexpression (35–37, 49, 52, 53). *PLAGL1* overexpression appears to reduce beta cell mass in neonates *via* its apoptotic and/or anti-proliferative capabilities (51). This is potentially due to PLAGL1-mediated modulation of *PPARG* and *PACAP1-R* expression, two key regulators of beta cell proliferation and insulin secretion, respectively (54). Additionally, high glucose levels reduced *Plagl1* expression in rodent beta cell lines and in primary mouse islets (55). *Plagl1* overexpression in several rodent beta cell lines impaired insulin secretion (56) and overexpression in mice recapitulates the early-onset diabetes observed in TNDM patients (57). Furthermore, induced *Plagl1* expression resulted in a decrease of glucose-stimulated proinsulin biosynthesis, despite an increase in insulin mRNA (55) suggesting that *Plagl1* can also negatively regulate the translational apparatus and ultimately the efficiency of insulin mRNA translation.

### Rasgrf1

*Rasgrf1* is a paternally expressed imprinted gene that functions as a guanine nucleotide exchange factor for Ras GTPases (58). Pancreatic islets from the *db/db* diabetic mouse model had a significant reduction in *Rasgrf1* expression (59). Mice with deletion of *Rasgrf1* showed reduced beta cell proliferation and neogenesis, and thus decreased beta cell mass, resulting in hypoinsulinaemia and impaired glucose tolerance (60). Interestingly, it was found that *Rasgrf1* is a direct *Plagl1* target gene in multiple rodent beta cell lines and in mouse islets (56). Moreover, a two-fold overexpression of *Plagl1* in beta cells resulted in repression of *Rasgrf1* expression and impaired insulin secretion, which could be rescued by restoring *Rasgrf1* expression (56).

### *Cdkn1c* and *Kcnq1*

*Cdkn1c* is expressed solely from the maternal allele (61, 62) and regulates cell proliferation and differentiation (63–65). Indeed, suppression of *CDKN1C* expression *via* viral delivery of shRNAs into isolated human islets provoked a 3-fold increase in beta cell proliferation and was sufficient to rescue hyperglycaemia when transplanted into diabetic mice (66). Importantly, newly-replicated beta cells retained the characteristics of mature beta cells, with expression of key functional markers (insulin, *PDX1* and *NKX6.1*) and a robust response to high glucose in terms of calcium dynamics (66). Also found at the *CDKN1C*-containing 11p15/ICR2 imprinted region, the *KCNQ1* gene encodes a voltage-gated potassium channel, and overexpression of this protein in mouse MIN6 beta cells causes impaired insulin secretion (67). Furthermore, administration of a KCNQ1 inhibitor enhanced insulin secretion in isolated islets and in mice (68). Genetic disruption of the *Kcnq1* gene in mice caused a reduction in beta cell mass and subsequent glucose intolerance, although this was likely due to a subsequent upregulation of *Cdkn1c* expression (69).

The incidence of hypoglycaemia is approximately 50% in patients with Beckwith-Wiedemann Syndrome (caused by genetic disruption at 11p15) and is associated with beta cell hyperplasia and subsequent hyperinsulinaemia in affected individuals (70–74) [reviewed in (75)]. In many Beckwith-Wiedemann Syndrome patients, the ICR2 region at 11p15 is hypomethylated on both alleles (76), causing loss of expression of *CDKN1C* (77) and is linked with an increase in proliferation of beta cells (66, 76, 78, 79). Furthermore, targeted demethylation of ICR2 using a methylcytosine dioxygenase 1 (TET1)-based approach repressed *CDKN1C* expression in human islets, with subsequent increased levels of Ki-67 and significant beta cell proliferation (80). Interestingly, a point mutation in the *CDKN1C* gene was found in a family with several features consistent with IMAGe syndrome (81), a growth and developmental disorder similar to Beckwith-Wiedemann Syndrome (82, 83), as well as an early-adult-onset form of diabetes (81). It remains to be determined whether IMAGe patients also develop diabetes at a later stage in life; however, these findings suggest that mutation of *CDKN1C* alone may be sufficient to drive a monogenic form of diabetes.

### *Dlk1* and *Gtl2/MEG3*

The imprinting region on human chromosome 14q32 carries a cluster of imprinted genes, including the paternally expressed gene *DLK1* and the maternally expressed long non-coding RNA (lncRNA) *MEG3* (*Gtl2* in rodents) (84–86). Overexpression of *Dlk1* in mice improves glucose tolerance and whole-body insulin sensitivity (87), potentially by promoting proliferation and differentiation of beta cells (88). Transgenic mice overexpressing *Dlk1* in pancreatic beta cells demonstrate an increase in islet mass with higher proportion of larger islets, whereas *Dlk1* null mice showed the opposite trend (89). Transgenic mice, therefore, had increased insulin secretion and improved glucose tolerance (89), although conversely, a different group has demonstrated increased proliferation (and size) of pancreatic islets upon *Dlk1* ablation in mice (90). At the same locus, constituent deletion of *Gtl2* and its associated promoter in mice led to severe parent-of-origin-dependent peri-/postnatal developmental defects and early lethality (91). Increased methylation at the *Gtl2* promoter DMR in the mouse beta cell line, βTC6, resulted in decreased *Gtl2* expression and increased beta cell sensitivity to cytokine-mediated oxidative stress (92). *Gtl2* has also been shown to maintain the expression of *Mafa*, a critical beta cell transcription factor that positively influences insulin synthesis and secretion (93). *Gtl2* expression is also decreased in islets in the *db/db* diabetic mouse model and its expression is glucose-regulated in both the MIN6 mouse beta cell line and in primary mouse islets (94). Knockdown of *Gtl2* using siRNA in both MIN6 beta cells and primary islets impaired insulin synthesis and secretion and caused beta cell apoptosis (94). Furthermore, knockdown of *Gtl2 in vivo* resulted in impaired glucose tolerance and insulin secretion in mice, likely due to a reduction in beta cell mass (94).

### Grb10

*Grb10* functions *via* intracellular signalling pathways regulating growth and metabolism (95) and has been implicated in binding to, and negatively regulating signals from, the insulin receptor (IR) and insulin-like growth factor 1 receptor (IGF1R) (96, 97). Differential transcriptome analysis of mouse islets from diabetes-resistant (ob/ob) *vs* diabetes-sensitive (New Zealand obese, NZO) mouse strains revealed a number of human diabetes candidate genes, including *Grb10* (98). Several studies have also uncovered a role for *Grb10* in the regulation of glucose handling; however, this has required picking apart the relative contribution from *Grb10* expression in the beta cell and other peripheral, insulin target, tissues (99–101). Disruption of *Grb10* expression in mice is associated with postnatal overgrowth and enhanced insulin secretion and sensitivity and improved glucose tolerance (99–103). Conversely, *Grb10* overexpression in mice caused postnatal growth retardation, accompanied by severe insulin resistance and worsened glucose intolerance (104, 105). Moreover, pancreatic-specific *Grb10* deletion resulted in increased beta cell proliferation and a subsequent increase in insulin content and secretion, and improved glucose tolerance (106). However, Prokopenko et al. found that shRNA-mediated knockdown of GRB10 in isolated human islets led to a reduction in insulin secretion (107).

## Evidence of Altered Imprinted Gene Expression in T2D

T2D is predominantly a consequence of beta cell failure (108, 109) and the majority of the genes associated with T2D pathogenesis encode modulators of beta cell function (110). The studies described above demonstrate that careful control of imprinted gene expression is required in order to maintain normal beta cell function and glucose homeostasis. Interestingly, several studies have shown that the expression and methylation patterns of several imprinted genes show notable differences in T2D *vs* non-diabetic islets (86, 107, 111–114) (summarised in **Table 1**). Specifically, *Dlk1* expression was found to be elevated in beta cells from patients with T2D (113) and has been posited as a biomarker for identifying women at high risk of developing diabetes (119). *MEG3* expression was also found to be downregulated in islets from T2D donors as a result of hypermethylation at the *MEG3* DMR (86). Additionally, a study consisting of patients with gestational diabetes mellitus showed that DNA methylation at the *MEG3*-DMR was positively correlated with maternal glycaemia and foetal growth (120). Using a genome-wide association study (GWAS) based on assessment of GSIS, Prokopenko et al. (107) demonstrated that inheriting variants of *GRB10* were associated with reduced GSIS and an increased risk of T2D when inherited from the father, but improved glycaemia when inherited from the mother, which may be due to the different parent-of-origin tissue expression patterns of *Grb10* (121, 122). Several studies have also identified single nucleotide polymorphisms (SNPs) at multiple imprinted *loci* associated with T2D and impaired glucose tolerance including those at the *CDKN1C* locus (amongst others) (115), *KCNQ1* (116, 117) and *GRB10* (118) (**Table 1**). Indeed, several imprinted genes including *Plagl1*, *Dlk1*, *Gtl2* and *Nnat* were differentially expressed between a ‘responder’ subclone of mouse MIN6 beta cells (based on their sustained GSIS capacity) *vs*. ‘non-responder’ beta cells (123). In the above scenarios, where the diabetic state is associated with altered DNA methylation and misexpression of imprinted genes in pancreatic beta cells, a major question centres around the temporal nature of these events. Are the observed changes in DNA methylation at key regulatory genomic regions acting as a primary driver of imprinted gene misexpression (and therefore functional changes) in these beta cells? Or do changes in nutrient status lead to misexpression of imprinted genes *via* other mechanisms (e.g. nutrient-specific transcription factors) that are later reinforced by long term epigenetic changes such as DNA methylation? A recent study has shown that even mild hyperglycaemia in rodents is sufficient to evoke deregulation of critical genes for beta cell identity, including *Nnat* (46), and it will be interesting to further explore this model in terms of epigenetic alterations longitudinally over periods of chronic, albeit mild, hyperglycaemia.

**Table 1:** Imprinted gene candidates for conferring susceptibility to type 2 diabetes.

Study	Imprinted *loci* or gene affected	Methods used	Human population(s)
(115)	11p15 and 7q32	GWAS - SNP chips (T2D *vs* control)	Icelandic
(116)	*KCNQ1*	GWAS - SNP genotyping (T2D *vs* control)	Japanese, Korean, Chinese and European
(117)	*KCNQ1*	GWAS - SNP genotyping (T2D *vs* control)	Japanese, Singaporean and Danish
(118)	*GRB10*	GWAS - SNP array (T2D *vs* control)	Amish and Scandinavian
(107)	*GRB10*	Meta-analysis of multiple GWAS (based on reduced GSIS) and SNP arrays	Multiple backgrounds
(86)	*MEG3*	Micro RNA sequencing in dispersed/FACS-sorted human islets (T2D *vs* control)	Multiple backgrounds
(113)	*DLK1* and *PLAGL1*	Single cell transcriptomics in dispersed human islets (T2D *vs* control)	Multiple backgrounds
(112)	*PEG3*	RNA and exome sequencing in whole human islets (T2D *vs* control)	European
(111)	*KCNQ1*	Genome-wide DNA methylation and transcriptomic analysis in dispersed/FACS-sorted human islets (T2D *vs* control)	Swedish
(114)	*GRB10*	Genome-wide DNA methylation and transcriptomic analysis with SNP array in isolated human islets from non-diabetic donors	Swedish

This has been assessed using GWAS and SNP analysis or via differential expression and/or methylation of imprinted genes in isolated islets from T2D vs control subjects.

## Metabolic Programming and T2D in the Next Generation

**Figure 2:**
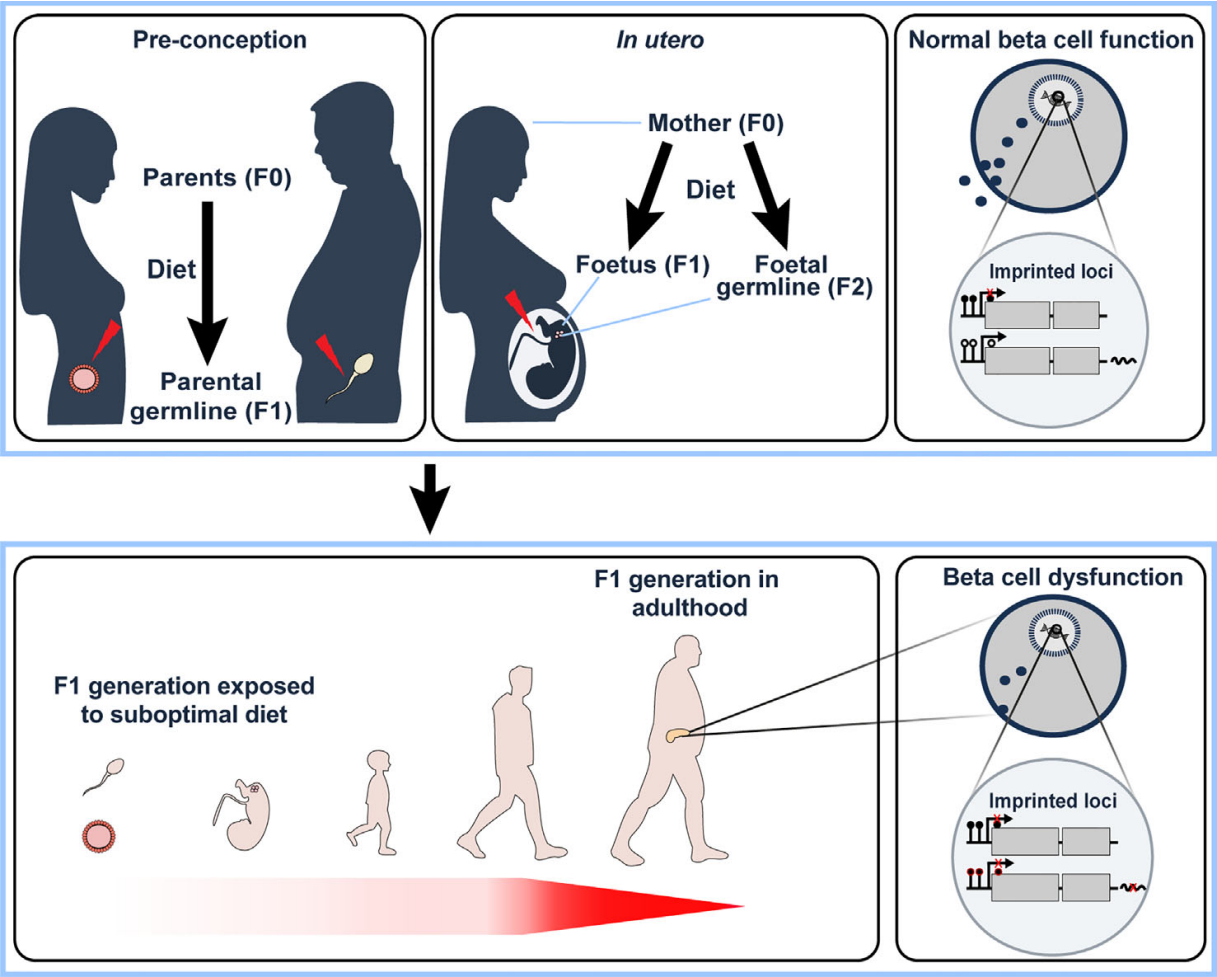
Under- or overnutrition influences imprinted gene expression not only in the individual (F0 generation) but also in the next generation (F1). This may occur indirectly *via* pre-conceptual changes in the germ cells (upper left panel) or *via* direct exposure *in utero* (and potentially the subsequent (F2) generation *via* direct exposure of foetal germ cells to nutritional alterations *in utero*, upper middle panel). F1 offspring that have been directly or indirectly exposed to a suboptimal nutritional status in early development have been shown to develop beta cell dysfunction in adulthood (lower left panel). In the F0 generation, overnutrition has been shown to alter imprinted gene expression in pancreatic beta cells *via* changes in DNA methylation at key regulatory genomic regions. We therefore hypothesise that changes in nutritional status affects the monoallelic expression of imprinted genes that is observed in ‘normal’ conditions (upper right panel) *via* alterations to CpG methylation, with an example illustrated in the lower right panel (closed circles – methylated CpGs, open circles – unmethylated CpGs). With their known functional role in beta cells, deregulation of imprinted gene expression *via* the diet would, therefore, lead to beta cell dysfunction. It will be interesting to determine the relative contribution of imprinted gene deregulation on the observed beta cell dysfunction in the F1 generation due to nutritional status in the F0 generation.

It is becoming increasingly apparent that individuals can be predisposed to adult-onset metabolic diseases, such as T2D, due to the direct effect of their nutritional status in early development, either *in utero* or in the first few years of life (124, 125). The developmental origin of health and disease (DOHaD) hypothesis, first put forward by Barker (126, 127) suggested that exposure to environmental factors during vulnerable periods of foetal development or early childhood might increase an individual’s risk to metabolic disease in later life; this has since been linked with possible mediation by epigenetic factors (1, 128–133). Indeed, intrauterine growth restriction (IUGR) or parental overnutrition in rodents and humans results in impaired glucose homeostasis in adulthood (134–140). One of the first examples of this phenomenon was the finding that children born during the Dutch Winter Famine of 1944-45, who were exposed to maternal undernutrition *in utero*, went on to develop diabetes in later life (141) with evidence for altered DNA methylation at imprinted *loci* (142, 143). Moreover, it has been suggested that the transfer of epigenetic changes to the next generation are not limited to exposure to the developing foetus (i.e. *in utero* nutrition), but also directly to gametic cells, with evidence for altered DNA methylation at imprinted *loci* in oocytes from diabetic female mice (144). Furthermore, chronic paternal high-fat diet feeding, prior to conception, in rodents leads to impaired insulin secretion and glucose tolerance in their offspring, including altered expression of imprinted genes (145), indicating that both these epigenetic changes and beta cell dysfunction can be passed on to the next generation *via* the male germline. Similar findings have also been documented in children who were conceived by obese fathers, with evidence for altered expression (and methylation) at imprinted loci (*IGF2*, *PEG3*, *MEG3*, *PLAGL1* and *NNAT*) in F1 offspring (146, 147) (**Figure 2**).

## Conclusions and Final Perspectives

The expression of imprinted genes is heavily influenced by epigenetic mechanisms such as DNA methylation. Multiple lines of evidence demonstrate that imprinted genes are critical for beta cell function and that they are nutritionally regulated in these cells. Misexpression of imprinted genes is associated with both rodent models of diabetes and T2D islets, with evidence that altered methylation and/or expression at these *loci* by the diet can be passed on to the next generation either *in utero* or *via* gametic cells. Using imprinted gene *loci*, with their well understood epigenetic control and functional importance in beta cells, will help us to understand the type and genomic distribution of epigenetic marks that are established in response to overnutrition. Indeed, the plasticity of the epigenome enables both a flexibility in response to environmental factors (e.g. diet) and also a potential target for epigenetic-modifying drugs that may be used to enhance insulin secretion. Epigenetic editing at imprinted *loci* has already been shown to be a promising tool to promote beta cell expansion (80) and epidrugs directed as specific molecular targets e.g. methyltransferases, that preserve beta cell functional identity during periods of suboptimal nutritional status, represent an exciting therapeutic possibility for T2D. We therefore need a better understanding of the diet-induced epigenomic changes responsible for misexpression of imprinted (and non-imprinted) genes that negatively impact beta cell function. This would enable us to test the ability of specific epidrugs to target and inhibit these pathways in beta cells in the face of nutrient excess. Indeed, if modification of epigenetic status at imprinted *loci* is proven to be a reliable biomarker for reduced beta cell function, this approach could be employed to assess the effect of specific dietary components/macromolecule content on insulin secretion in model systems. A key question for the future is also whether any epigenomic changes observed at the beta cell level are preserved in other cells (e.g. blood cells, subcutaneous adipose tissue) that can easily be sampled from patients. In this scenario, we could harness these molecular alterations to better predict future diabetic outcomes in patients and intervene in disease progression prior to long term hyperglycaemia and beta cell failure.

## Author Contributions 

All authors contributed to the article and approved the submitted version. Writing – Original Draft Preparation, CV-H and SM. Writing – Review and Editing, CV-H and SM.

## Funding

SM was supported by a Wellcome Trust/Imperial College ISSF Fellowship (PS3619_WREC).

## Conflict of Interest

SM has received a speaker honorarium from the Society for Endocrinology.

The remaining author declares that the research was conducted in the absence of any commercial or financial relationships that could be construed as a potential conflict of interest.
